# Pharmacokinetic Studies of Antisense Oligonucleotides Using MALDI-TOF Mass Spectrometry

**DOI:** 10.3389/fphar.2020.00220

**Published:** 2020-03-25

**Authors:** Markus Herkt, Ariana Foinquinos, Sandor Batkai, Thomas Thum, Andreas Pich

**Affiliations:** ^1^Hannover Medical School, Institute of Molecular and Translational Therapeutic Strategies, Hanover, Germany; ^2^Hannover Medical School, Institute for Toxicology – Core Unit Proteomics, Hanover, Germany

**Keywords:** microRNAs, therapeutic oligonucleotides, mass spectrometry, absolute quantification, pharmacokinctics

## Abstract

Cardiac diseases are the most frequent causes of death in industrialized countries. Pathological remodeling of the heart muscle is caused by several etiologies such as prolonged hypertension or injuries that can lead to myocardial infarction and in serious cases also the death of the patient. The micro-RNA miR-132 has been identified as a master-switch in the development of cardiac hypertrophy and adverse remodeling. In this study, MALDI-TOF mass spectrometry (MS) was utilized to establish a robust and fast method to sensitively detect and accurately quantify anti-microRNA (antimiR) oligonucleotides in blood plasma. An antimiR oligonucleotide isolation protocol containing an ethanol precipitation step with glycogen as oligonucleotide carrier as well as a robust and reproducible MS-analysis procedure has been established. Proteinase K treatment was crucial for releasing antimiR oligonucleotides from plasma- as well as cellular proteins and reducing background derived from biological matrices. AntimiR oligonucleotide detection was achieved from samples of studies in different animal models such as mouse and pig where locked nucleic acids-(LNA)-modified antimiR oligonucleotides have been used to generate pharmacokinetic data.

## Introduction

MALDI-TOF mass spectrometry (MS) has been widely used for the analysis of many different molecules including oligonucleotides. Its advantages lie in efficient and fast sample preparation and precise analysis using highly resolving reflector TOF systems. Due to its negatively charged backbone, DNA is difficult to stabilize. Cobalt (III) hexamine and ammonium citrate dibasic have been utilized to stabilize DNA during desorption (Distler and Allison, 2002; Sudha and Zenobi, 2002). Natural and modified oligonucleotides were characterized by MALDI-MS and the use of 2,4,6-trihydroxy acetophenone (THAP) as ionization matrix. Di- and triammonium salts of organic and inorganic acids were utilized to remove various ion adducts and prevent peak widening (Pieles et al., 1993). An ionization matrix of 3,4-diaminoparabenzophenone (DABP) has been proposed to yield lower detection limits, less fragmentation and less ion adducts especially at low oligonucleotide concentrations. In addition, samples were highly homogenous resulting in excellent shot-to-shot reproducibility, resolution, and low background noise (Fu et al., 2006).

Extraction of analytes from an organic matrix is mandatory for analysis of oligonucleotides. One of the least complex methodologies is reducing the background noise by diluting samples. Several approaches use a proteinase K digestion or methanol precipitation to remove proteins from the biological samples with a recovery of oligonucleotides reaching 98% (Bellon et al., 2000; Waters et al., 2000; Arora et al., 2002). In addition, many approaches include a phenol/chloroform extraction (Griffey et al., 1997; Beverly et al., 2006). For oligonucleotide extraction, chemical extraction using denaturing agents followed by organic solvent precipitation and solid-phase extraction by immobilizing the RNAs on a glass support are two classical approaches (Beverly et al., 2006).

As for oligonucleotides, molecular masses can indicate modifications and composition. However, sequence informations are not available. This analysis can be performed by digestion of oligonucleotides with subsequent analysis of fragmentation products by MS analysis (Pomerantz and McCloskey, 2005). MS-based fragmentation techniques as for instance in-source decay (ICD) of digested oligonucleotides yield ions that are typical for base and sugar fragments of the nucleoside and allow further localization of the modification in the structure of the parent ion. With this nuclease digestion sequencing and partial sequencing of larger oligonucleotides can be accomplished. For this approach, aliquots from the digestion reaction are measured at specific time intervals. By using either a 3′ or a 5′ specific exonuclease, approximately 3–4 bases from each end can be determined (Hossain and Limbach, 2007).

The fragments arise from cleavages along the phosphodiester backbone resulting in a series of ions that can be used to determine the order within an oligonucleotide. Secondary fragmentation of large oligonucleotides resulting in more complex MS/MS-spectra or a different fragmentation behavior due to backbone modifications, complicates further analysis (Pomerantz and McCloskey, 1990; Bartlett et al., 1996). Derivatization is one way to enhance fragmentation behavior that occurs due to the intrinsic character of these sugar or nucleobase modifications (Emmerechts et al., 2005). The detection limit is still the most challenging issue for analyzing oligonucleotides. Detection limit for single stranded DNA with a phosphorothioate modification was described at 4 ng/mL (Zhang et al., 2007). A method for relative quantification of tRNAs was described where tRNAs were isotopically labeled resulting in quantification in the femtomole range (Castleberry and Limbach, 2010).

Sufficiently low detection limits are still the most challenging issues in oligonucleotide analysis besides efficient separation and ionization efficiency. Finding the fine balance between separations and obtaining high signal intensity requires further research. The analytical detection of oligonucleotides will be a major point to facilitate effective research and clinical applications of oligonucleotides.

Cardiac disorders are amongst the leading causes of morbidity and mortality worldwide. Adverse remodeling causes contractile dysfunction and the failing of the heart that will lead ultimately to the patient’s death (Hill and Olson, 2008). One of the central mechanism in the remodeling process is cardiomyocyte hypertrophy. At the molecular level, the FoxO transcription factor family suppresses calcineurin/NFAT signaling and thus functions as an anti-hypertrophic agent due to induction of autophagy and apoptosis in cardiomyocytes (Ni et al., 2006; Glas, 2010; Ronnebaum and Patterson, 2010). The miR-212/132 family was identified as a master-switch in the development of cardiac hypertrophy and adverse remodeling. MiR-132 is upregulated due to myocardial stress resulting in cardiac hypertrophy *in vivo* and pathological hypertrophy of cardiomyocytes *in vitro* (Ucar et al., 2012). Administration of miR-132 inhibitors (antimiRs) inhibited the development of cardiac hypertrophy mimicking the effects seen in miR212/132 KO mice (Ucar et al., 2012). Thus, miR-132 seems to be as a promising target in heart failure drug development.

The combination of MALDI and a time-of-flight (TOF) mass analyzer (MALDI-TOF) is widely used to analyze many different analytes, especially oligonucleotides. Therefore, the aim of this study was the development of a novel, fast, and reliable MALDI-MS-based technique for quantification of therapeutic oligonucleotides to enable pharmacokinetic studies of therapeutic oligonucleotides in mouse and pig.

## Materials and Methods

### Chemicals and Reagents

All chemicals and reagents are listed and described below in alphabetical order (Table 1). For all substances the purity is at least p.a. or higher. Purity of LNA-modified oligonucleotides containing a phosphorothioate backbone was determined by *EXIQON* with HPLC-ESI-MS analysis (Table 2).

**TABLE 1:** Chemicals and reagents used in this study.

Substance	Specifications
AA matrix-solution	10 mg/mL; 5 mg/mL L-Fucose in 70% methanol *Sigma-Aldrich Fluka*
ACN	*Merck*
Acryl-/Bisacrylamid	*Serva Electrophoresis GmbH*
AHC solution	300 mg/mL; *Sigma-Aldrich*
ATT matrix-solution	5 mg/mL; 5 mg/mL L-Fucose in 100% H_2_O *Sigma-Aldrich*
Bradford reagent	*Sigma-Aldrich*
CaCl_2_	dehydrated; *Fluka*
CHCA matrix-solution	10 mg/mL; 5 mg/mL L-Fucose in 50% ACN; 0.1% TFA; *Bruker Daltonics*
DABP matrix-solution	15 mg/mL; 5 mg/mL L-Fucose in methanol/HCl: 80/3 v/v; *Sigma-Aldrich*
DHB matrix-solution	20 mg/mL; 5 mg/mL L-Fucose in 50% ACN *Bruker Daltonics*
EDTA	*Sigma-Aldrich*
Ethanol	*Millipore*
Fixation solution	25% isopropanol; 10% acetic acid; 65% Millipore water
L-Fucose	*Fluka*
glycogen	20 μg/μL; *Invitrogen*
HPA matrix-solution	50 mg/mL; 5 mg/mL L-Fucose in 50% ACN *Bruker Daltonics*
Laemmli buffer	100 mM Tris–HCl, pH 8.8; 10% SDS (w/v); 100 mM DTT; 3% glycerol; 2 mg/mL Bromphenol blue
Methanol	*Merck*
NaCl	*chem*^*solute*^
PageBlue protein staining solution	*Thermo Fisher scientific*
Page Ruler^TM^ prestained protein ladder	10 – 250 kDa; *Fermentas GmbH*
Proteinase K Gold^TM^	0.2 g; A ≥ 30 U/mg protein (Hemoglobin, pH 7.5, 37°C); isolated from *Tritirachium album*; RNase-, DNase-, Exonuclease- free; *peqlab*
Proteinase K digestion buffer	10 mM Tris pH 8.0; 10 mM EDTA pH 8.0; 100 mM NaCl
Proteinase K solution	40 mg/mL proteinase K; 50 mM Tris pH 8.0; 10 mM CaCl_2_
SDS-separating gel (12%)	4 mL 30%/0.8% Acryl-/Bisacrylamid (w/v); 2.5 mL 1.5 M Tris–HCl, pH 8.8; 3.35 mL Millipore water; 100 μL 10% SDS (w/v); 50 μL 10% APS (w/v) (Serva); 5 μL TEMED
SDS-stacking gel (5%)	1.67 mL 30%/0,8% Acryl-/Bisacrylamid (w/v); 2.5 mL 0.5 M Tris–HCl, pH 6.8; 5.69 mL Millipore water; 100 μL 10% SDS (w/v); 50 μL 10% APS (w/v); 10 μL TEMED
SDS-running buffer	25 mM Tris–HCl, pH 8.3; 192 mM glycin; 0.1% SDS (w/v)
THAP matrix-solution	40 mg/mL; 5 mg/mL L-Fucose in 90% ACN *Sigma-Aldrich Fluka*
Tris	*MP Biomedicals*
Tris–HCl buffer	1.5 M; pH 6.8/8.8
Water	Distilled; *Millipore*

**TABLE 2:** Oligonucleotides used in this study.

Substance	Specifications
antimiR oligonucleotide Scr (scrambled)	Seq: ACGTCTATACGCCCA; *m*_*average*_ = 5016.1 Da; ε = 143100 L/mol*cm; purity > 85%; *EXIQON*
antimiR oligonucleotide 24	Seq: CTGCTGAACTGAGCC); *m*_*average*_ = 5044.0 Da; ε = 135900 L/mol*cm; purity > 85%; *EXIQON*
antimiR oligonucleotide 132	Seq: ATGGCTGTAGACTGTT; *m*_*average*_ = 5364.2 Da; ε = 154400 L/mol*cm; purity > 85%; *EXIQON*

### Sample Preparation From Complex Matrices for MS-Analysis

In this study murine, as well as porcine EDTA-plasma was used. As an internal standard 1.5 μL of a 200 pmol/μL antimiR oligonucleotide Scr, or 132 solution were added to 28.5 μL of plasma to achieve a final concentration of 10 pmol/μL. For Proteinase K digestion 30 μL of plasma containing antimiR oligonucleotides were mixed with 40 μL proteinase K solution and 330 μL Proteinase K digestion buffer. The mixture was incubated at 55°C with 450 rpm on an *Eppendorf Thermomixer comfort* for 16 h overnight. Subsequently, antimiR oligonucleotides were precipitated by adding 100 μg of glycogen and 2 volumes ethanol followed by incubation for 4 h at −20°C. Then the samples were centrifuged at 21100 x g for 30 min and 4°C with a *HERAEUS FRESCO 21 centrifuge*. Supernatant was removed and pellets were dissolved in 30 μL ammonium citrate dibasic (AHC) solution at RT.

### MALDI-TOF: Method for MS-Analysis

For MALDI-mass spectrometry (MALDI-MS) analysis a 5800 MALDI-TOF/TOF mass spectrometer (Sciex) was used. A special metal polish paste provided by Sciex successfully removed even the slightest impurities on the MALDI target plate so that a homogenous crystallization behavior of the analyte/matrix mix and thus a higher reproducibility of the analytical results was ensured. 5 μL of an antisense oligonucleotide (ASO) solution were mixed thoroughly with 5 μL of THAP matrix-solution in a 0.5 mL tube by vortexing with subsequent spotting of 0.5 μL of this solution per spot onto an MALDI target plate. This added a dilution factor of four on spot in terms of molarity. The Acquisition method was set up to the following parameters: *Fixed Laser Intensity*: 6100; *Mass Range (Da)*: 4.000 to 6.000; *Focus Mass*: 5.000; *Delay Times (ns)*: 600; *X2 Deflector*: 0.055; *Shots/sub-spectrum*: 200; *Total Shots/Spectrum: 3000*; *Stage Velocity* (μ*m/s)*: 600; *Bin Size (ns)*: 1.0; *Vertical Offset (% full scale)*: −0.5; *Detector Voltage Multiplier*: 0.67; *Laser Pulse Rate (Hz)*: 400. The other parameters were set to standard. For analysis the *TOF/TOF*^TM^ *Series Explorer*^TM^ Software Version 4.1.0 (build 12), Oracle Database Schema Version 4.0.0, Data Version 4.0.4 was used.

### Quantification of antimiR Oligonucleotides

For quantification of oligonucleotides, peak areas were utilized. For each analysis, three technical replicates per sample were generated and the values were averaged with subsequent determination of standard deviation (SD) as well as the coefficient of variation (CV).

The limit of detection (LOD) and lower limit of quantification (LLOQ) of antimiR oligonucleotides in mice as well as pig plasma were determined by setting up several dilutions of antimiR132. In addition, antimiR*Scr* was added to each sample at a final concentration of 10 pmol/μL as an internal standard. Afterward 5 μL of antimiR oligonucleotide solution were mixed thoroughly with 5 μL of THAP matrix-solution (Table 1) in a 0.5 mL tube by vortexing with subsequent spotting of 0.5 μL of this solution per spot on an MALDI target plate. MS analysis was performed as described above. Ratios obtained from antimiR*132* and antimiR*Scr* were inserted into the calibration curve equation (Figure 3), which was then resolved to x to obtain the amount of substance. Taking into account all dilution factors, the concentration of the substance was then calculated.

### Hannover Mouse PK Study

All experimental procedures involving mice were performed in accordance with the local and national regulations and approved by local animal welfare bodies of the Hannover Medical School, Germany or University of Kaposvar, Hungary. Male C57BL/6N Crl mice were administered once at the beginning of the experiment with 20 mg/kg antimiR132 intravenous (i.v.) and sacrificed after various time points to remove plasma and organs, namely heart and liver. The respective substance and dose combination was applied intravenously by administration into the lateral tail veins. For blood collection, the mouse was placed under anesthesia using isoflurane. The study plan is depicted in Table 3.

**TABLE 3:** Mouse PK study plan.

Intervention	Substance	Dosing	Number of animals per time of blood collection							Σ
			3 min	30 min	1 h	9 h	24 h	3 days	1 weeks	
i.v.	antimiR*132*	20 mg/kg	3	3	3	3	3	3	3	21
Control	Control	Control	3	3	3	3	3	3	3	21

Additionally, heart and liver were taken 24 h, 72 h, and 1 weeks post application of antimiR*132*.

### Pilot PK Study, in Life Phase

The tissue and plasma samples for this assay development study were derived from an animal study and used in all described experiments in different quantities and specification.

#### Animal Groups and Size

The study was conducted in female domestic pigs (Danish Landrace bread, 29 – 31 kg body weight), using three experimental groups. There was a treated group of three animals, which were injected with antimiR132. A placebo group (Placebo) of three animals was injected with 0.9% physiological saline. A control group of three animals received no treatment.

#### Treatment and Sample Collections

AntimiR132 and placebo was injected as a 20 mL solution via cardiac, intracoronary (i.c.) perfusion (80 mL/h for 15 ± 5 min) in three concentrations: 0.5 mg/kg (low dose), 1.17 mg/kg (medium dose), and 5 mg/kg (high dose). The treatment procedure was carried out on day 0 under general anesthesia following a coronary angiography. Blood samples were taken from both treated as well as untreated group. On day 1 organs and blood samples were taken from both groups before i.c. application (baseline) and at 15 min, 30 min, 1 h, 6 h, 12 h, and 24 h post treatment. After last blood sampling was completed, animals were euthanized and organs and blood samples were taken. Control animals were also euthanized and tissue and blood (only one) samples collected. Blood samples were collected in EDTA and serum tubes and processed within 1 h post collection. The tissue samples were collected at study end-point, cut into pieces and snap frozen on dry ice. Tissue and aliquots of blood samples were stored at −80°C until analysis. The study plan is depicted in Table 4.

**TABLE 4:** Pig PK study plan.

Parameters			EDTA plasma						Organs									
Intervention	Substance	Dosing	15 min	30 min	1 h	6 h	12 h	24 h	Heart	Liver	Kidney	Brain	Lung	Spleen	Ovary	Skin	Fat	Muscle
i.c.	antimiR*132*	0.5 mg/kg	X	X	X	X	X	X	X	X	X	X	X	X	X	X	X	X
i.c.	antimiR*132*	1.17 mg/kg	X	X	X	X	X	X	X	X	X	X	X	X	X	X	X	X
i.c.	antimiR*132*	5 mg/kg	X	X	X	X	X	X	X	X	X	X	X	X	X	X	X	X
i.c.	NaCl	–						X	X	X	X	X	X	X	X	X	X	X
Control	Control	Control						X	X	X	X	X	X	X	X	X	X	X

### Bradford Protein Assay

The protein concentration of mouse plasma was determined by Bradford protein assay. Plasma or tissue extracts were diluted with Millipore water if necessary and 5 μL were mixed with 200 μL of Bradford reagent (Table 1) and 800 μL of Millipore water with subsequent incubation for 5 min at RT. Afterward absorbance levels were determined at λ = 595 nm in triplicates. Protein concentration was then calculated for the obtained absorbance levels based on the calibration curve done with bovine serum albumin (Supplementary Figure 1).

### SDS-PAGE

Protein samples (50 – 100 μg) were mixed with 0.25 volumes of Laemmli buffer (Table 1) and incubated for 10 min at 95°C. If necessary, proteins were alkylated by addition of 1 μL of a 40% acrylamide solution and incubation at RT for 30 min. Proteins were applied to a 12% Acryl-/Bisacrylamid SDS-gel (Table 1) and separation was performed with a SDS-running buffer (Table 1) and a 5% stacking gel (Table 1) for 15 min at 100 V and 1h at 180 V. The Page Ruler^TM^ Prestained Protein Ladder (Table 1) was used. After separation gels were washed 3 times with Millipore water for 10 min with subsequent treatment with a fixation solution (Table 1). Staining was performed with 20 mL PageBlue Protein Staining Solution (Table 1). Destaining was done 3 times with 200 mL Millipore water each for 15 min.

## Calculation

An example for the quantification of antimiR132 in porcine Plasma 1 h post high dose (5 mg/kg) treatment is shown below:

(1) y=0.4033⁢x⁢(Calibration⁢curve⁢equation⁢for⁢plasma)

Converted to x this results in equation (2)

(2) x=y/0.4033

With y = 0.194 the result is equation (3)

(3) x=0.194/0.4033=0.481⁢pM

Multiplied by the dilution factor of 4 (derived from 1:1 mixture with the THAP matrix-solution and that only 0.5 microL were applied on spot) results in equation (4):

(4) c=4x=40.481*=1.924pmol/μL

## Results

### Initial Studies

Six matrices were prepared: 9-aminoacridine (AA), α-cyano-4-hydroxycinnamic acid (CHCA), 2,5-dihydroxybenzoic acid (DHB), 6-Aza-2-thiothymine (ATT), 3-hydroxypicolinic acid, 2,4,6-Trihydroxyacetophenone (THAP), and 2,5-diaminobenzophenone (DABP) (Table 1). In addition, a final concentration of 5 mg/mL L-Fucose was added to every matrix solution. Every matrix was then mixed with 30 pmol/μL antimiR oligonucleotide *24* and spotted on a MALDI target plate and analyzed. Analysis was performed in linear negative- as well as in linear positive detection mode for all matrix/antimiR oligonucleotide solutions. Reflector detection mode and linear positive detection mode did not yield any evaluable results. Only HPA and THAP could show evaluable results in linear detection mode. Best signal intensities were obtained using THAP in linear negative detection mode (Supplementary Figure 2). These results indicated best ionization efficiency for THAP as ionization matrix and thus it was decided to use THAP for further analysis.

**FIGURE 1:**
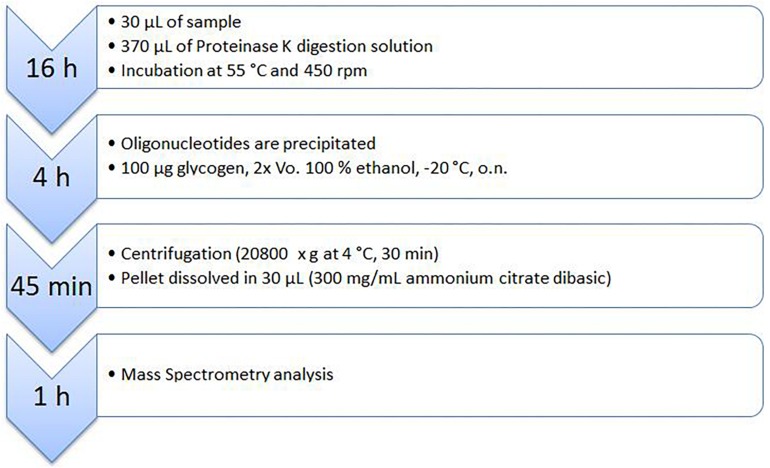
Workflow of antimiR oligonucleotide detection using MALDI-MS.

Full extraction of an analyte from a biological sample is crucial for quantification. Since phosphorothioate modified oligonucleotides were reported to strongly bind to plasma proteins as well as cell surface proteins, a proteinase K digestion was used to digest those proteins and thus releasing oligonucleotides and decreasing the background noise originated from complex matrix components (Beltinger et al., 1995; Geary et al., 2001). The workflow consisted of two main steps: a) protein digestion at 55°C using proteinase K and b) glycogen-facilitated precipitation of the oligonucleotide. Subsequently, oligonucleotides were measured by MALDI-MS (Figure 1). Effectiveness of protein removal is shown in Supplementary Figures 3, 4.

For extraction of antimiR oligonucleotides from complex matrices four steps were necessary. With being the most important step, Proteinase K digestion was followed by precipitation of antimiR oligonucleotides with subsequent resuspension and MS analysis.

**FIGURE 2:**
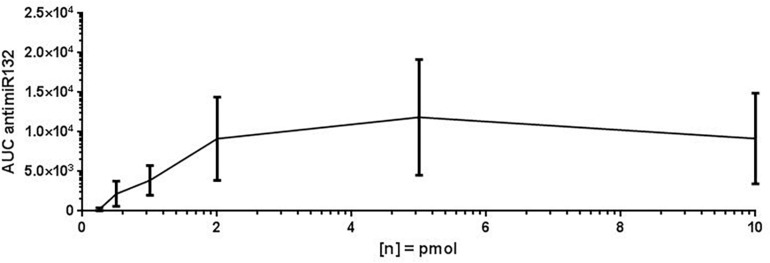
Limit of detection (LOD) of antimiR132. For determination of the LOD, antimiR oligonucleotides were spotted on a MALDI target plate after isolation from murine plasma. Analyses were done in 10 replicates and the peak area (=signal intensity) were plotted against concentration. The LOD is 0.5 pmol.

**FIGURE 3:**
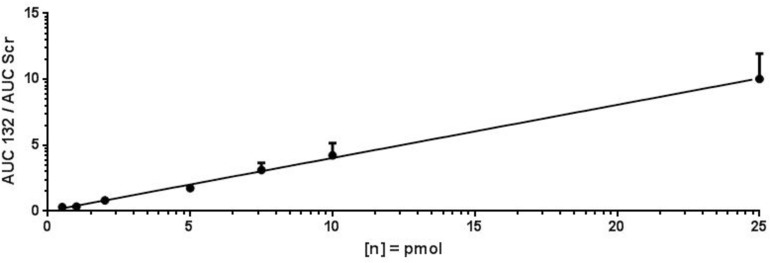
LLOQ and area of quantification of antimiR132. For determination of the LLOQ and AOQ with MALDI-MS, antimiR oligonucleotides were spotted into murine plasma, extracted, and analyzed by MALDI-MS in three replicates per oligonucleotide concentration with the LLOQ being at 0.25. The equation of the calibration curve was y = 0.4013x + 0.0331 with a coefficient of determination of 99.77%. The LLOQ is indicated by a red box.

For assay validation purposes, the LOD, LLOQ and area of quantification (AOQ) values for detection of antimiR132 were determined. Validation of the isolation protocol was performed by adding antimiR132 at various concentrations (10, 5, 2, 1, 0.5, 0.25, and 0.1 pmol) together with 10 pmol antimiRScr as internal standard to murine plasma from untreated mice and measured with the developed assay. Analysis was performed with 10 replicates for determination of the LOD being at 0.25 pmol and three replicates for determination of the LLOQ and the AOQ. The LLOQ was determined to be at 0.25 pmol. AOQ was determined to be linear in the range from 0.25 up to 25 pmol (Figures 2, 3).

### *In vivo* Studies

**FIGURE 4:**
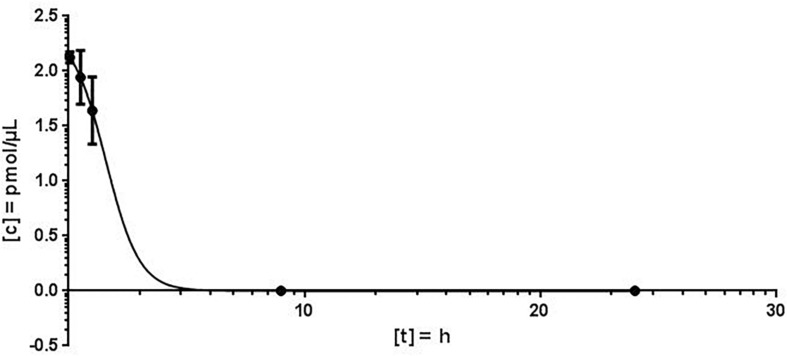
Murine plasma concentration of antimiR*132*. After application of 20 mg/kg antimiR132 i.v., mouse plasma levels were assessed utilizing MALDI-MS.

For the quantification of antimiR132 in murine plasma, 20 mg/kg antimiR132 was delivered i.v. to 21 mice. At seven timepoints (3 min; 30 min; 1 h; 9 h, 1 days; 3 days; 1 week) blood samples were taken from which 100 – 200 μL plasma were obtained. AntimiRScr was added as an internal standard in a final concentration of 10 pmol/μL. By increasing the NaCl concentration from 100 mM to 250 mM in the Proteinase K buffer, a higher efficiency of the enzymatic reaction was achieved. The quantification of antimiR oligonucleotides was carried out as described previously. The plasma concentrations determined for each time were averaged over all three biological replicates with the standard deviation and the coefficient of variation (CV) being determined (Figure 4).

**FIGURE 5:**
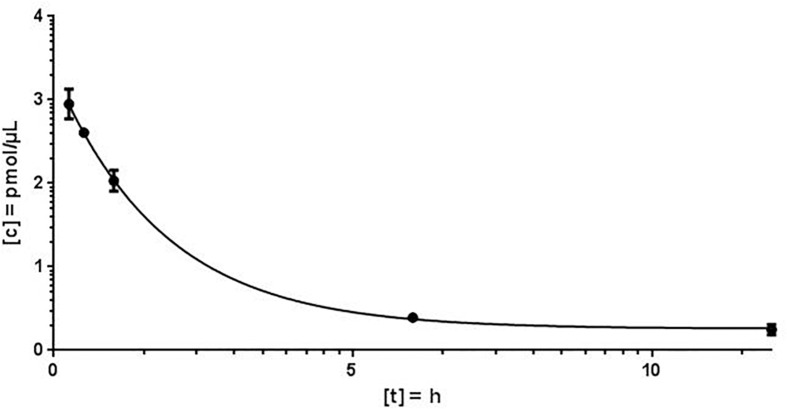
Porcine plasma concentration of antimiR132. After application of 5 mg/kg antimiR132 i.c., pig plasma levels were assessed using a mass spectrometry-based method for quantification. CV = 3.6 – 24.2%.

Using the MALDI-MS analysis, a plausible kinetic could be determined. The coefficient of variation (CV) varied from 2.4 to 18.7% and the peak concentration was calculated to 2.124 pmol/μL plasma. The plasma concentration decreased rapidly and plasma half-life of antimiR132 could be determined to be approximately 2 h. However, at least 9 h post treatment no antimiR132 was detected (Figure 5).

To evaluate the pharmacokinetic properties of antimiR132, further blood plasma samples from pig taken 15 and 30 min; 1, 6, 12, and 24 h after application were examined for the presence of antimiR132 and subsequently quantified (Figure 5).

Using MALDI-MS, a plausible kinetic could be determined. The peak concentration could be calculated at 2.124 pmol/μL. As expected, the plasma concentration decreased rapidly with the plasma half-life of antimiR132 being after approximately 2 h with no longer detection after 9 h. The concentration fell below the LOD and LLOQ after 12 h (Figure 4).

## Discussion

An antimiR oligonucleotide isolation protocol as well as a robust and reproducible MALDI-MS analysis procedure has been established. Proteinase K digestion was the important releasing antimiR oligonucleotides bound to plasma and cellular proteins. MALDI-MS analysis in linear negative detection mode with THAP as ionization matrix was proven to be best suited for oligonucleotide analysis in MALDI-MS and highly sensitive and reproducible values for antimiR132 could be determined. The high salt conditions might have reduced the binding affinity of LNA-based antimiR132 to plasma proteins. With this experimental setup the LOD and LLOQ for antimiR132 were determined and showed high sensitivity of this approach. The LOD as well as the LLOQ were at 0.25 pmol (1 pmol/μL) while the AOQ ranged to 25 pmol (100 pmol/μL, allowing a wide range for quantification (Figures 2, 3). These results show that MALDI-MS is a very sensitive and highly specific method for derivatized oligonucleotides of interest. That is less than is achieved with LC-MS approaches where reported sensitivity was 10 fmol/μL assuming for an oligonucleotide 5000 g/mol (Gallo-Cantafio et al., 2016). However, the LOD and LLOQ values are at an acceptable low range to quantify effective concentrations that are used in model systems to test efficacy of oligonucleotides as pharmacological agent (Tuntland et al., 2014). The advantage of MALDI-MS is its easy handling and very fast analysis times that allow measurements of e.g., 100 samples in about 15 min.

With MALDI-MS, a mouse PK study with antimiR132 was performed in which mice were treated i.v. with 20 mg/kg of this substance and sacrificed afterward with blood and organs taken. After reaching the peak concentration 3 min after application the plasma levels decreased strongly and antimiR132 could not be detected in the plasma after 9 h (Figure 4). In addition to the qualitative detection of antimiR132 in pig plasma, pharmacokinetic parameters were also obtained (Figure 5). After 12 h antimiR132 was no longer quantitatively detectable. Accordingly, the latter was either renally excreted or absorbed by the tissue. This relatively short plasma half-life of antimiR132 was to be expected since earlier studies with similar antimiR oligonucleotides also showed a similar trend even across species and support the assumption that plasma clearance of phosphorothioate-modified oligonucleotides might be species independent since plasma half-lifes were comparable (Yu et al., 2009).

## Conclusion

MALDI-MS is a very precise and quick method for quantification of modified antisense oligonucleotides. Despite being mostly used for qualitative analysis, MALDI-MS can also be utilized for quantitative purposes, if sample size is adequate. It is more straightforward compared to other approaches such as ESI-MS, since sample preparation time is noticeable reduced and a liquid chromatography system is not needed, which further reduces preparative effort. However, its sensitivity is high enough to detect lower amounts of antimiR oligonucleotides in blood samples or maybe tissues.

## Data Availability Statement

All datasets generated for this study are included in the article/Supplementary Material.

## Ethics Statement

The animal study was reviewed and approved by the Local Animal Welfare Bodies of the Hannover Medical School, Hannover, Germany and the University of Kaposvar, Kaposvár, Hungary.

## Author Contributions

MH, AF, SB, TT, and AP conceived and performed the experiments, and wrote the manuscript. AP and TT secured the funding. SB, AP, and TT provided the expertise and feedback.

## Conflict of Interest

TT is founder and shareholder of Cardior Pharmaceutical. He has filed and licensed patents about noncoding RNAs. SB is an employee of Cardior Pharmaceuticals.

The remaining authors declare that the research was conducted in the absence of any commercial or financial relationships that could be construed as a potential conflict of interest.
