# Supplementary material for: Pharmacokinetic Studies of Antisense Oligonucleotides Using MALDI-TOF Mass Spectrometry
Source: Front Pharmacol. 2020 Mar 25;11:220. doi: 10.3389/fphar.2020.00220 (PMC7109322; doi:10.3389/fphar.2020.00220)
Supplement: Supplementary file 1 [file Data_Sheet_1.PDF]

Pharmacokinetic Studies of Antisense Oligonucleotides Using MALDI-TOF Mass Spectrometry

M. Herkt[1], A. Foinquinos[1], S. Batkai[1], T. Thum[1]*, A. Pich[2]*

[1]Institute of Molecular and Translational Therapeutic Strategies (IMTTS), Hannover Medical School (MHH), Hannover, Germany

[2]Institute for Toxicology – Core Unit Proteomics, Hannover Medical School (MHH), Hannover Germany

Keywords: microRNAs; therapeutic oligonucleotides; mass spectrometry; absolute quantification

*Joint senior

Correspondence should be addressed to herkt.markus@mh-hannover.de

Hannover Medical School, Institute of Molecular and Translational Therapeutic Strategies
Carl-Neuberg-Str. 1, 30625 Hannover, Lower Saxony, Germany
Tel: 0049-511-532-9174
Email: herkt.markus@mh-hannover.de

Hannover Medical School, Institute for Toxicology, Core Unit Proteomics
Carl-Neuberg-Str. 1, 30625 Hannover, Lower Saxony, Germany
Tel: 0049-511-5614
Email: pich.andreas@mh-hannover.de

Supplementary material

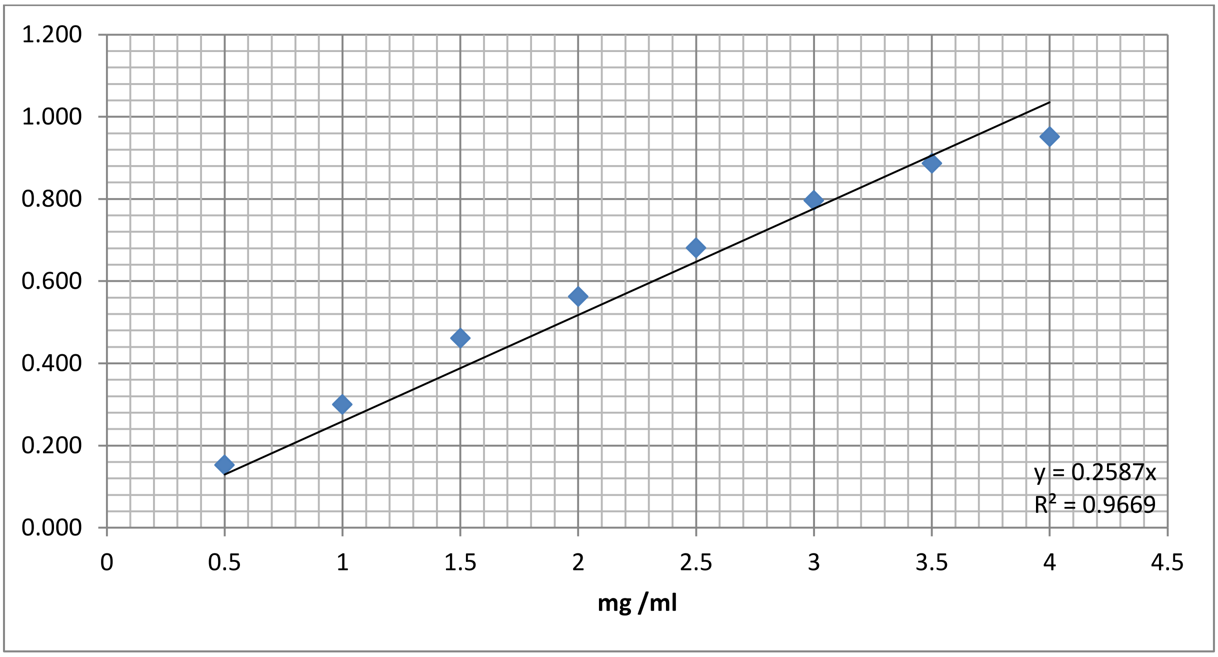

Supplementary Figure 1

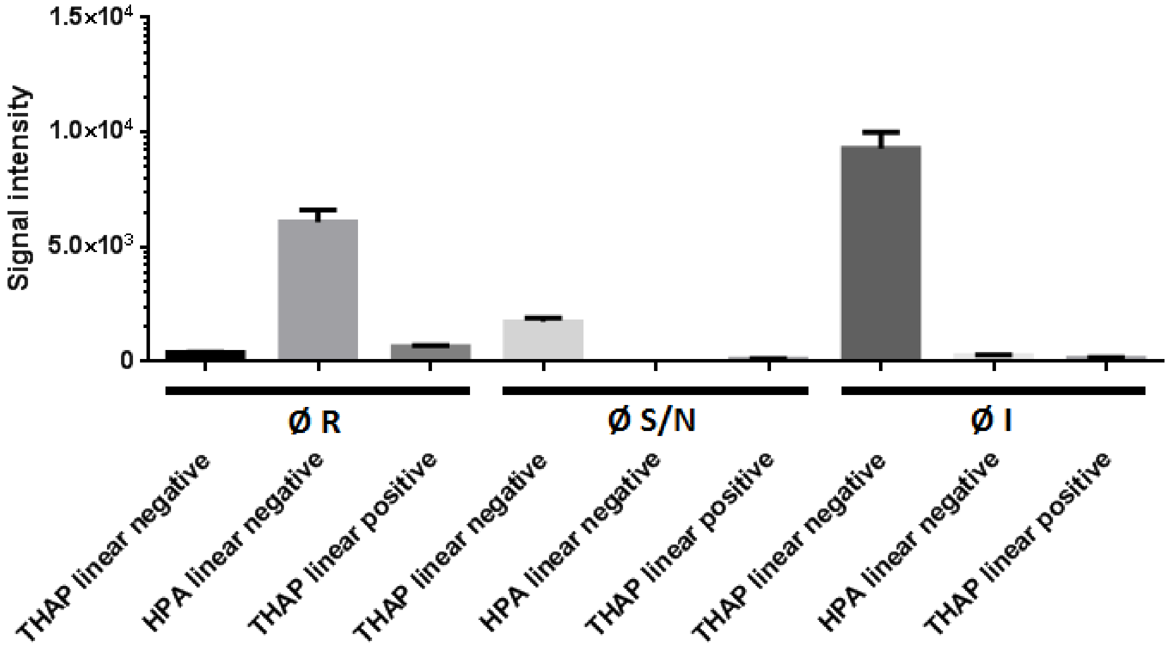

Supplementary Figure 2

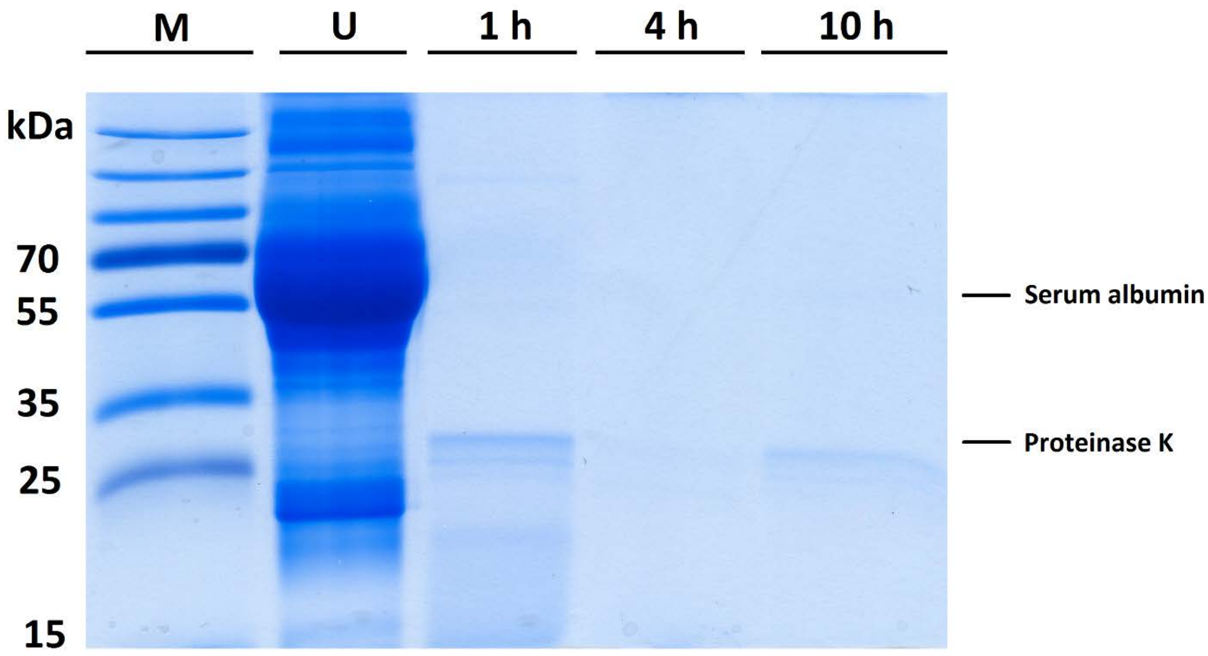

Supplementary Figure 3

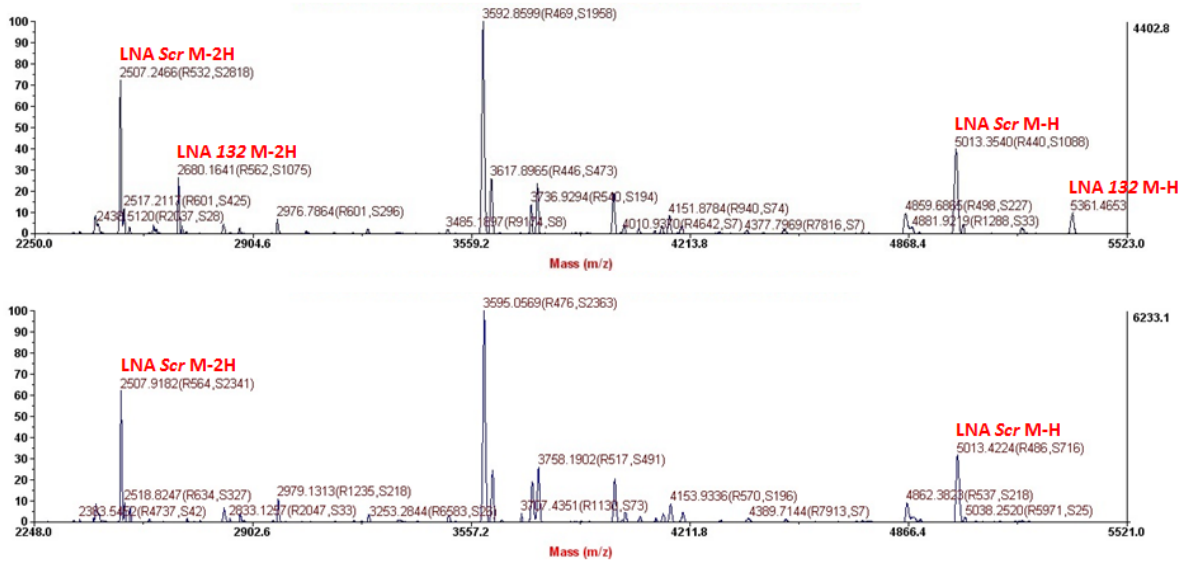

Supplementary Figure 4

Supplementary Figure 1| Calibration curve for Bradford protein assay. Samples were diluted to yield absorption levels between 0.1 and 1.0.

Supplementary Figure 2| Comparison of matrices for ionization. For determination of ionization-efficiency 30 pmol/µL of antimiR oligonucleotide *24* were mixed with different matrices and subsequently spotted on target. Analysis was performed with six technical replicates. Only THAP and HPA showed proper ionization of antimiR oligonucleotide *24* with best ionization for THAP. Ø R: mean resolution; Ø S/N: mean signal to noise ratio; Ø I: mean intensity.

Supplementary Figure 3| SDS-Page of digestion kinetics. 50 µg untreated and digested protein per lane were applied, respectively. M: Protein ladder (**Table 1**); U: untreated whole cell lysate.

Supplementary Figure 4| Mass spectra of porcine plasma samples. Male pigs were injected with 5 mg/kg antimiR*132* i.c. (high dose) and sacrificed after 24 h. The figure shows mass spectra of plasma isolates. antimiR*Scr* was added in a concentration of 10 pmol/µL as an internal standard. AntimiR*132*-specific peaks could be observed 24 h after injection. Upper panel: Mass spectra from high dose pig; Lower panel: Mass spectra from sham pig (negative control. AntimiR*Scr*/132 M-H: mono-charged species; AntimiR*Scr*/*132* M-2H: dual-charged species.
